# Metagenome next-generation sequencing plays a key role in the diagnosis and selection of effective antibiotics on the treatment of *Nocardia* pneumonia: a case report

**DOI:** 10.3389/fmed.2024.1373319

**Published:** 2024-05-27

**Authors:** Na Fan, Huang Fang, Fang Huang, Jie Zhou, Peng Liu, Meng-Jie Li, Ye-Ying Ding

**Affiliations:** ^1^Department of Respiratory Oncology, Renmin Hospital of Qingxian, Cangzhou, China; ^2^Department of Neurology, General Hospital of Central Theater Command of the People’s Liberation Army, Wuhan, China; ^3^Department of Laboratory Medicine, Renmin Hospital of Qingxian, Cangzhou, China; ^4^Department of Infectious Diseases, Hospital 902 of Joint Logistics Support Force of People’s Liberation Army, Bengbu, China

**Keywords:** *Nocardia*, *otitidiscaviarum*, immunocompetent patient, next-generation sequencing (NGS), pulmonary co-infection

## Abstract

*Nocardia* disease is an opportunistic infection, the occurrence is rare and mostly occurs in patients with immune deficiency. Even if the patient is immunocompetent, it can still be life-threatening. This case report describes a previously healthy 78-year-old male farmer with lung lesions discovered on a computerized tomography scan. Combined with the patient’s history of fever and the results of elevated laboratory markers associated with inflammation, the patient was diagnosed with a lung infection. After escalating empirical broad-spectrum antibiotics, antiviral and antifungal therapy, the patient continued to deteriorate to septic shock. In the meanwhile, the patient’s sputum was cultured repeatedly, and no obvious positive pathogenic bacteria were found. Considering the patient was elderly and that these lesions were solid with burr signs, as well as the progression after antimicrobial therapy cancer was considered in the differential diagnosis. Artificial intelligence (YITU, Hangzhou Yitu Medical Technology Limited Company) was also applied, and it also calculated that these lesions were cancerous. The patient received a puncture biopsy of the largest lung lesion. During the puncture pus was withdrawn from largest lung lesion. Culture and metagenome next-generation sequencing (mNGS) detection performed on pus indicated *Nocardia otitidiscaviarum*. The test report of the mNGS is also attached with a susceptibility report of commonly used clinical antibiotics to this *Nocardia* spp. Using this result, the patient’s disease was quickly controlled after selecting the targeted drug compound sulfamethoxazole and intravenous meropenem for treatment. In view of the high misdiagnosis rate and poor sensitivity of culture for *Nocardia* spp., this case emphasized mNGS playing a key role in the diagnosis and selection of effective antibiotics for the treatment of *Nocardia* spp. lung infections.

## Introduction

*Nocardia* spp. is an aerobic species of prokaryotic microorganism that is widely found in soil, air, spoilage plants, and other organic matter (1). To date, 791 *Nocardia* spp. isolates were identified, of 119 recognized *Nocardia* spp. species with valid names, 54 were related to human infection among the existing literatures (2). The *Nocardia* nova complex, *Nocardia cyriacigeorgica*, *Nocardia farcinica* are the most common pathogenic bacteria, and the cases of *Nocardia otitidiscaviarum* as pathogenic bacteria are few (3). *Nocardia* is generally considered to be an opportunistic pathogen that causes infection in people with immune dysfunction, such as long-term oral hormone medications, diseases associated with malignant tumors and/or organ transplantation, and HIV patients (4). Infections caused by *Nocardia* spp. often manifest as subacute, chronic localized, or disseminated suppurative infections (5, 6). Infection by *Nocardia* spp. is most commonly acquired by inhalation of bacteria into the lungs (5, 6). Subsequent haematogenous spread to the central nervous system and other sites can then occur (7). A low index of clinical suspicion and the low percentage of positive cultures for *Nocardia* spp. infection may lead to a misdiagnosis (8). The resultant delayed appropriate treatment may have disastrous consequences. *Nocardia* lung abscess is easily misdiagnosed as lung cancer in clinical practice, especially when the disease continues to progress after antibiotic treatment in some patients (9, 10). We report a case of a feverish patient with lung lesions that were suspected to be lung cancer after the disease progressed despite anti-inflammatory and antibiotic therapy. Enlightening, metagenome next-generation sequencing (mNGS) indicated *Nocardia otitidiscaviarum*, which provided an accurate result for the differential diagnosis.

## Case report

**FIGURE 1:**
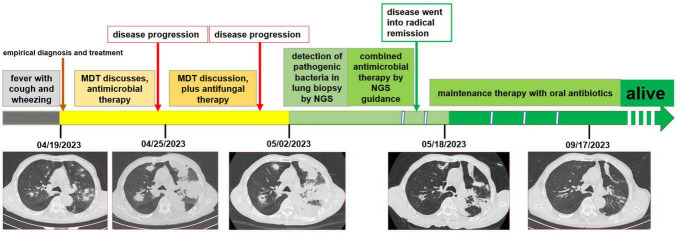
The timeline of the patient’ disease evolution and treatments. **(A)** On admission; **(B)** six days after admission; **(C)** thirteen days after admission; **(D)** one month after admission; **(E)** five months after admission.

A 78-year-old male farmer was previously in good health. This admission to hospital was due to fever with cough and wheezing for two weeks. The patient denied a history of diabetes mellitus or immune system disease, and had no history of food or drug allergy, travel, or pet contact. Two weeks prior, he had a cold with a fever. His highest body temperature was 38.5°C. Subsequently, the patient developed a cough with sputum, which was yellow and easily produced at a rate of about 10 ml per day. Physical examination at hospital presentation showed that the temperature was 37.7°C, the pulse rate was 110 beats/min, and the respiration rate was 20 breaths/min, the blood pressure was 143/94 mmHg. The patient was alert and wheezing. The superficial lymph nodes in the axilla, groin, neck, and clavicle regions were not large, and a few moist rales could be heard in both lungs. The rhythm of the heart was irregular, and premature beats could be heard. The abdomen was flat and soft, the liver and spleen were not palpable, and there was no edema of both lower limbs. The patient received a computerized tomography (CT) scan examination of chest, which revealed multiple solid lesions in both lungs (the largest is about 4 cm in diameter), small pleural effusion in the left thorax, and mediastinal adenopathy (see Figure 1A). The results displayed elevated laboratory markers (white blood cell count (neutrophile granulocyte), C-reactive protein, erythrocyte sedimentation rate) associated with inflammation (see Table 1). The other laboratory results such as blood biochemical tests and blood lung cancer markers showed no special anomalies (see Table 1).

**TABLE 1:** Patient’s laboratory test results.

Laboratory tests	Normal range	Results
WBC (×109/L)	4∼10	19.34
Neutrophile granulocyte (×109/L)	2∼7	18.12
Neutrophile granulocyte percentage (%)	50–70	93.7
C-reactive protein (mg/L)	< 10	203
Albumin (g/L)	0∼40	24.5
Alanine aminotransferase (U/L)	7∼40	36
Aspartate aminotransferase (U/L)	13∼35	19
Direct bilirubin (umol/L)	0∼6.8 μmol/L	5.2
Creatinine (umol/L)	41∼81	50.1
Urea nitrogen (umol/L)	3.6∼9.5	4.61
Erythrocyte sedimentation rate (mm/h)	0∼20	128
CA125 (U/mL)	0∼35	17.50
CA19-9 (U/mL)	0∼37	6.84
CYFRA21-1 (ng/mL)	0∼3.3	1.62
SCC (ng/mL)	0∼1.5	1.65
NSE (ng/mL)	0∼16.3	8.49
CEA (ng/mL)	0∼5	2.19
HbA1c (%)	4.0∼6.0	4.7
Protein (Urinalysis routine)	Negative	0
Urobilinogen (Urinalysis routine)	Negative	0
Occult blood (Urinalysis routine)	Negative	0

Combined with the patient’s history of fever and the results of elevated laboratory markers associated with inflammation, the patient was diagnosed with a lung infection by the multidisciplinary team (MDT) discussion. Therefore, the patient was treated with intravenous moxalactam sodium as antibiotic treatment and intravenous diprophylline for antiasthmatic treatment and symptomatic supportive treatment. One week after the treatment, the patient’s symptoms worsened and thick yellow sputum appeared. In the meanwhile, the patient’s sputum was cultured repeatedly, and no obvious positive pathogenic bacteria were found. After the second MDT discussion it was suggested to perform G-(1,3)-β-D -glucan test (G test), galactomannan test (GM test), and Cryptococcus testing. It was suggested that pulmonary fungal infection or viral infection should be considered in the case of poor response to antibacterial therapy. Intravenous meropenem combined with voriconazole and ribavirin were then given. Subsequent tests, such as G test, GM test, cryptococcal antigen test were all negative.

Chest CT reexamination was performed, which revealed a markedly enlarged bilateral area of patchy hyperdense opacities, with large patches of ground-glass opacities surrounding the lesions (see Figure 1B). Considering the patient was elderly and that these lesions were solid with burr signs, as well as the progression after antimicrobial therapy cancer was considered in the differential diagnosis. Artificial intelligence (YITU, Hangzhou Yitu Medical Technology Limited Company) was also applied, and it also determined that these lesions were cancerous. The third MDT discussion resulted in the suggestion that these lesions may be lung cancer with intrapulmonary metastasis. The infection was considered to be part of obstructive pneumonia caused by cancer. The patient underwent fiberoptic bronchoscopy, which revealed no abnormalities in the bronchi other than thick sputum. The pathogen detection by cultured of duct lavage fluid also did not obtain significant results. After one week of treatment and failure of tests to find the cause, the patient continued to progress to septic shock.

**FIGURE 2:**
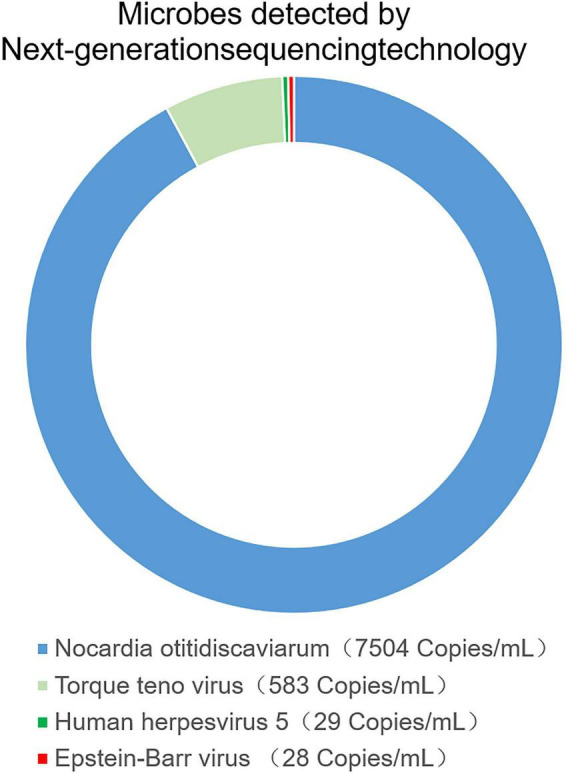
The pus was found in percutaneous lung neoplasm biopsy puncture, which was detected microbes by metagenome next-generation sequencing technology (mNGS).

**FIGURE 3:**
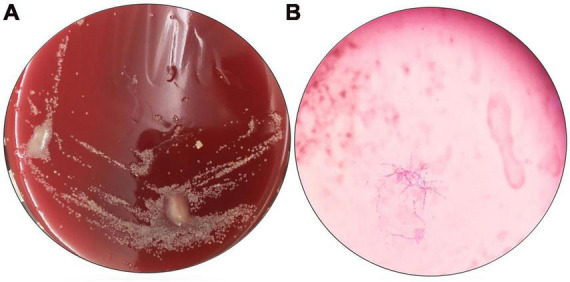
Results of pathogen culture in the pus. **(A)** Culture original by nutrient agar; **(B)** oil mirror of pathogenic bacteria by weak anti-acid staining, 1000×.

The patient received a chest CT re-examination (see Figure 1C) and a puncture biopsy of the largest lung lesion under CT guidance. During the puncture pus was withdrawn from largest lung lesion. Culture and metagenome next-generation sequencing (mNGS) were performed on the pus and the puncture fluid. Next-generation sequencing (NGS) was conducted on the pus specimen. Various microbes were detected by mNGS (see Figure 2), which showed *Nocardia otitidiscaviarum* was the pathogenic bacterium (the *Nocardia otitidiscaviarum* probe sequence is F- ACCGACCGAGATATTCAAATGATGA, R- TTCCACGAAAGGAAAGAACAATGTT, Probes- CGAGTCCAGCTGCGCCGAGAGT). Culture of the puncture fluid was positive and displayed dry chalky white colonies (see Figure 3). At this moment, the patient was diagnosed with pulmonary infection by *Nocardia otitidiscaviarum*.

According to NGS testing guidance, compound sulfamethoxazole 1.5 g every 6 h was given orally combined with intravenous meropenem. Two days later, the patient’s temperature returned to normal, his spirits gradually improved, his cough decreased, and his sputum volume decreased. Ten days after this treatment, chest CT re-examination was performed, which revealed a reduction in the extent of bilateral lung shadow (see Figure 1D). The patient was scheduled to be discharged with oral trimethoprim-sulfamethoxazole for another 4 months. Subsequent follow-up re-examination of the chest CT showed that the lesions were absorbed in both lungs (see Figure 1E).

## Discussion

*Nocardia* spp. is filamentous actinobacteria of the order corynebacteriales and mostly known for their ability to cause life-threatening infections in humans (11). *Nocardia* spp. are responsible for a range of diseases that affect various organ systems, including the lungs, skin, soft tissue and the intracranial- and abdominal cavity (12, 13). The lungs are the primary site of infection, accounting for 75–80% of the total infection (14). With *Nocardia* spp. invading and causing pulmonary infections known as pulmonary nocardiosis (PN). PN is characterized by suppurative inflammation and necrosis within the lungs, triggered by the inhalation of *Nocardia* spp. spores or fragmented hyphal elements through the respiratory tract (1, 14).

PN often manifests as cough, dyspnea, fever, night sweats, anorexia, fatigue, weight loss, hemoptysis, and pleuritic chest pain (15). The imaging manifestations of pulmonary *Nocardia* spp. infection lack specificity and can be manifested as single or multiple nodules, pulmonary mass (with or without cavity), reticular nodular infiltration, interstitial infiltration, lobar consolidation, subpleural plaque and pleural effusion (16, 17). Imaging features like these can easily be misdiagnosed as lung cancer or viral pneumonia (18–20). This misdiagnosis is more common when empirical antimicrobial treatments have failed (21). Even if the diagnosis is more accurate with AI, there are still misdiagnoses of such lesions. Therefore, the diagnosis of PN is difficult.

At present, it is recognized that *Nocardia* spp. isolated from clinical specimens is the gold standard for the diagnosis of PN (22). However, due to its special biological characteristics, the positive detection rate of *Nocardia* spp. is low. And the positive detection rate of *Nocardia* spp. detected from sputum is low in PN patients (23). In addition, when the results of the culture are negative, they may have to be repeated multiple times (22, 23). Even so, no positive results were obtained from culture of peripheral blood, sputum and duct lavage fluid in this case. As mNGS does not require traditional microbial culture, nor does it require specific amplification, it is theoretically able to detect any pathogen with a known genome sequence. In addition to its high sensitivity, mNGS can detect pathogens even when their numbers are low due to its ability to sequence DNA at a sufficient depth. Compared to culture-based methods requiring an average feedback time of 7–14 days, mNGS provides pathogenic evidence within 2–3 days (21). Its ability to directly type pathogens without the need for further strain identification enables immediate initiation of targeted treatment based on the type of pathogen identified (24). As a result of this characteristic of mNGS more precise clinical use of drugs can be achieved and pathogen treatment can be optimized (25). Therefore, mNGS has transformed the diagnostic landscape of lower respiratory tract infections (LRIs) and the selection of effective antibiotics to treat pathogenic bacteria (26).

Treatment of this disease should follow the principles of adequate dose, adequate course of treatment and individualization. Drug susceptibility testing is particularly important as a guide. Literature data showed that PN is generally effective to sulfonamides antibiotics, and effective to linezolid, imipenem and amikacin (16, 17). Antimicrobial susceptibilities for *Nocardia* spp. have been shown to be species specific and may differ geographically, which highlights the importance of susceptibility testing (17, 25, 26). Therefore, this knowledge of antimicrobial susceptibilities for *Nocardia* spp. may highlight the utility of mNGS, which could suggest susceptibility and drugs that need to be excluded from use. The bacteria detected by mNGS can directly provide accurate clinical drug guidance, achieve targeted treatment of infected bacteria, and optimize the treatment plan (27). Combination therapy can enhance the efficacy and should be used as the initial treatment (28, 29). Common regimens include sulfamethoxazole combined with imipenem or amikacin, imipenem combined with cefotaxime or amikacin, and amikacin combined with cefotaxime. Triple therapy should be considered for immunocompromised patients with disseminated infection, and the course of treatment should be 6–12 months (30, 31).

In conclusion, PN is rare and occurs mostly in immunocompromised patients. The diagnosis of *Nocardia* spp. infection in immunocompetent individuals is often difficult. NGS of sputum, alveolar lavage fluid and lung tissue can improve the detection rate of *Nocardia* spp. infection and avoid catastrophic consequences. This case report has certain reference significance for the diagnosis and treatment of special diseases in the department of respiratory medicine and critical care medicine.

## Data availability statement

The original contributions presented in this study are included in the article/supplementary material, further inquiries can be directed to the corresponding author.

## Ethics statement

Ethical review and approval were not required for the study on human participants in accordance with the local legislation and institutional requirements. The patient provided written informed consent to participate in this study. Written informed consent was obtained from the individual for the publication of any potentially identifiable images or data included in this article.

## Author contributions

NF: Data curation, Formal analysis, Writing – original draft. HF: Funding acquisition, Project administration, Resources, Supervision, Writing – original draft, Writing – review & editing. FH: Data curation, Methodology, Writing – review & editing. JZ: Project administration, Validation, Writing – review & editing. PL: Data curation, Formal analysis, Project administration, Validation, Writing – original draft. M-JL: Data curation, Methodology, Writing – original draft. Y-YD: Conceptualization, Formal analysis, Funding acquisition, Investigation, Methodology, Project administration, Validation, Visualization, Writing – original draft, Writing – review & editing.
